# Effects and Safety of Calcimimetics in End Stage Renal Disease Patients with Secondary Hyperparathyroidism: A Meta-Analysis

**DOI:** 10.1371/journal.pone.0048070

**Published:** 2012-10-25

**Authors:** Qian Zhang, Ming Li, Li You, Haiming Li, Li Ni, Yong Gu, Chuanming Hao, Jing Chen

**Affiliations:** 1 Division of Nephrology, Huashan Hospital, Shanghai Medical College, Fudan University, Shanghai, China; 2 Department of Respiratory Medicine, Shanghai Tenth People’s Hospital Affiliated to Tongji University, Shanghai, China; 3 Division of Nephrology, Department of Medicine, Vanderbilt University Medical Center, Nashville, Tennessee, United States of America; University of Sao Paulo Medical School, Brazil

## Abstract

**Purpose:**

Secondary hyperparathyroidism (SHPT) is one of the most common abnormalities of mineral metabolism in patients with chronic kidney disease. We performed a meta-analysis to determine the effect and safety of cinacalcet in SHPT patients receiving dialysis.

**Methods:**

The meta-analysis was performed to determine the effect and safety of cinacalcet in SHPT patients receiving dialysis by using the search terms ‘cinacalcet’ or ‘mimpara’ or ‘sensipar’ or ‘calcimimetic’ or ‘R586’ on MEDLINE and EMBASE (January 1990 to February 2012).

**Results:**

Fifteen trials were included, all of which were performed between 2000 and 2011 enrolling a total of 3387 dialysis patients. Our study showed that calcimimetic agents effectively ameliorated iPTH levels(WMD, −294.36 pg/mL; 95% CI, −322.76 to −265.95, *P*<0.001) in SHPT patients and reduced serum calcium (WMD, −0.81 mg/dL; 95% CI, −0.89 to −0.72, *P*<0.001) and phosphorus disturbances(WMD, −0.29 mg/dL; 95% CI, −0.41 to −0.17, *P*<0.001). The percentage of patients in whom there was a 30% decrease in serum iPTH levels by the end of the dosing was higher in cinacalcet group than that in control group(OR = 10.75, 95% CI: 6.65–17.37, *P*<0.001). However, no significant difference was found in all-cause mortality and all adverse events between calcimimetics and control groups(OR = 0.86, 95% CI: 0.46–1.60, *P* = 0.630; OR = 1.30, 95% CI: 0.78–2.18, *P* = 0.320, respectively). Compared with the control therapy, there was a significant increase in the episodes of hypocalcemia (OR = 2.46, 95% CI: 1.58–3.82, *P*<0.001), nausea (OR = 2.45, 95% CI: 1.29–4.66, *P* = 0.006), vomiting(OR = 2.78, 95% CI: 2.14–3.62, *P*<0.001), diarrhea(OR = 1.51, 95% CI: 1.04–2.20, *P* = 0.030) and upper respiratory tract infection (OR = 1.79, 95% CI: 1.20–2.66, *P* = 0.004)in calcimimetics group.

**Conclusions:**

Calcimimetic treatment effectively improved biochemical parameters of SHPT patients receiving dialysis without increasing all-cause mortality and all adverse events.

## Introduction

Secondary hyperparathyroidism (SHPT) is one of the most common abnormalities of mineral metabolism in patients with chronic kidney disease (CKD), and is characterized by hyperplasia of the parathyroid glands and increased plasma levels of parathyroid hormone (PTH) [1]. It is well documented that disturbance in vitamin D, phosphorus, calcium and PTH metabolism contributes to bone disorders and cardiovascular complications of end stage renal disease patients and is associated with the morbidity and mortality of this population [2], [3].

The traditional treatment for SHPT is oral or intravenous administration of vitamin D sterols to lower PTH levels and (Ca- and non-Ca based) phosphate binders to control hyperphosphatemia. Although vitamin D sterols have been shown to be effective in suppressing elevation of serum PTH levels, they also increase serum P and Ca levels through stimulating gastrointestinal absorption [4], and therefore only a few patients were able to achieve the recommended therapeutic targets [5].

The calcium-sensing receptor (CaR) is a G protein–coupled cell-surface receptor that binds calcium ions and senses extracellular levels of calcium ion [6], [7]. The calcimimetic agents increase the sensitivity of CaR to extracellular Ca ion levels, leading to decreased PTH synthesis and secretion [8]. In 2004, the US Food and Drug Administration (FDA) approved cinacalcet (Sensipar) as the first calcimimetic drug for the treatment of SHPT. It improves PTH control without increasing circulating levels of calcium and phosphate [9]. The benefits and the adverse effects of calcimimetics vs. conventional therapy in dialysis patients with SHPT remain uncertain. The primary goal of this meta-analysis was to determine the effects and safety of cinacalcet in dialysis patients with SHPT.

## Patients and Methods

### Data Sources

**Figure 1:**
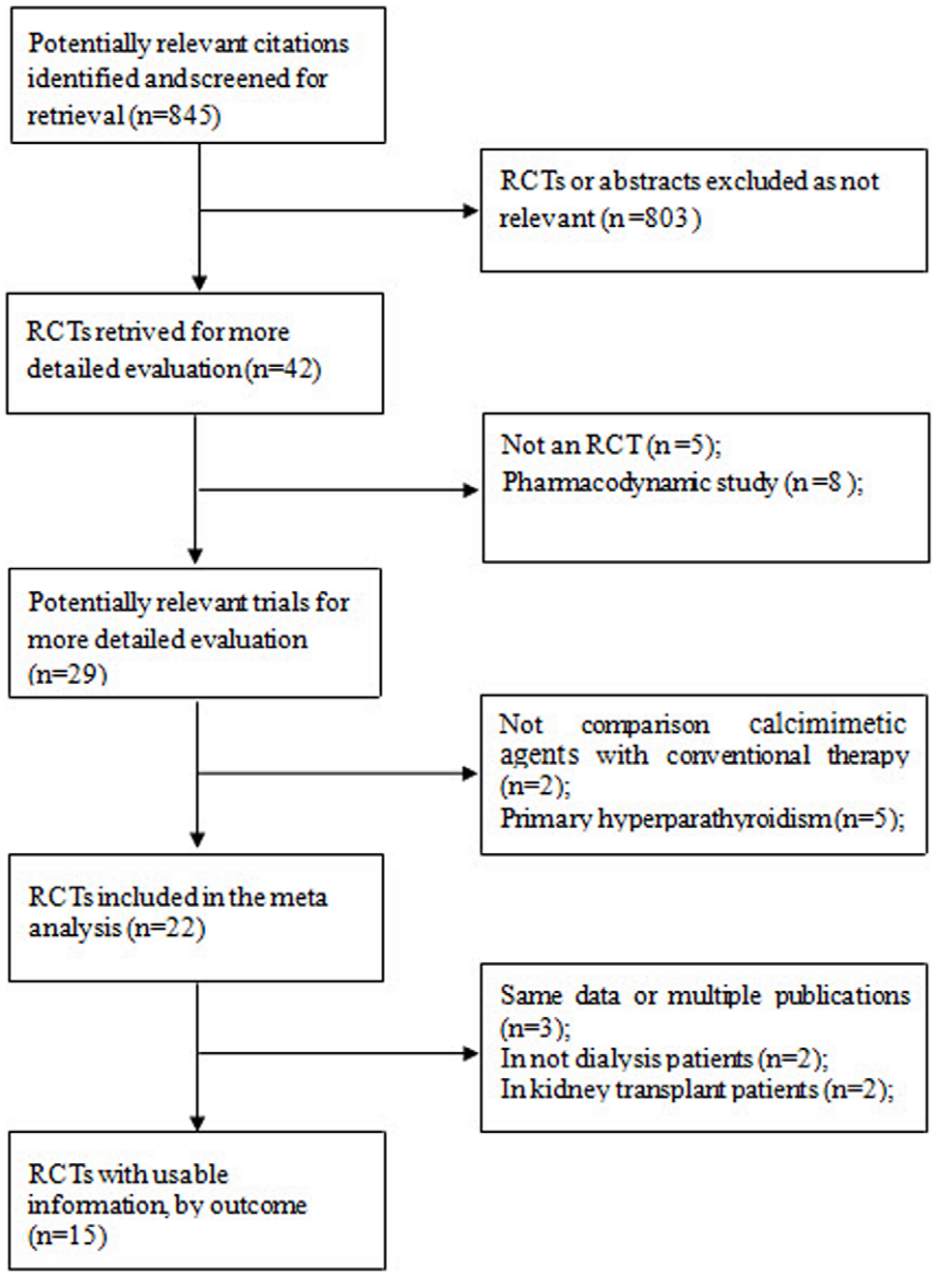
Procedure used for the trial selection. Abbreviation: RCT, randomized controlled trial.

A literature search was performed using the relevant search terms ‘cinacalcet’ or ‘mimpara’ or ‘sensipar’ or ‘calcimimetic’ or ‘R586’ on MEDLINE (January 1990 to February 2012) and EMBASE (January 1990 to February 2012) (Figure 1). These terms were also searched in the abstracts of conference proceedings of the American Society of Nephrology (ASN) between 1996 and 2011. Only randomized controlled trials that fulfilled the criteria of a highly sensitive filter were included in this study [10]. References of all included trials and review articles were scanned for additional studies.

### Study Selection

Study reports were included if they: (1) were randomized controlled trials; (2) enrolled adult human subjects undergoing dialysis and receiving calcimimetic agents or control treatment (placebo, conventional care); and (3) were clinical trials regardless of the publication status (published, conference proceedings, or unpublished), trial year, and language of publication. Two individuals independently inspected each reference and applied the inclusion criteria. For possibly relevant articles or in cases of disagreement, all authors would inspect the full article independently.

### Data Extraction and Quality Assessment

Two individuals independently extracted data from all primary studies that fulfilled the inclusion criteria, with disagreements resolved by consensus. For studies without reporting the outcome, the authors would be contacted for additional information. The same reviewers independently assessed trials for methodological quality using the Jadad scoring system [11], with disagreements resolved by consensus. The Jadad score is based on the explicit description of the study in the text as “randomized” and “double-blind”, and reporting of “withdrawals and dropouts”.

### Definition of Outcomes

The following biochemical outcomes were considered: values for intact PTH (iPTH), serum calcium level, serum phosphorus and calcium phosphorus product levels, bone alkaline phosphatase, osteocalcin and tartrate-resistant acid phosphatase. Patient-level outcomes included: all-cause mortality, all adverse events, hypocalcemia, nausea, vomiting, diarrhea, dyspnea, upper respiratory tract infection, and headache.

### Data Synthesis and Analysis

Data were analyzed using Review Manager (RevMan, Version 5.0, Copenhagen: The Nordic Cochrane Centre, The Cochrane Collaboration, 2008). Means and SDs were obtained for all continuous variables. When they were not available, they would be calculated them from data obtained from the investigators, from figures, or by recalculation from other effect estimates and dispersion measures [10]. Mean differences (MDs) were analyzed for continuous variables. Dichotomous data were compared using an odds ratio (OR). Respective 95% CI was calculated for each estimate and presented in forest plots. The statistical heterogeneity of trial results was assessed using the χ^2^ test for heterogeneity and the I^2^ test for inconsistency [12], [13]. If the *P* value was less than 0.1 (χ^2^ test), the results were considered heterogeneous; if the I^2^ was greater than 50%, the results were considered inconsistent [14]. If the test results for heterogeneity were significant, the DerSimonian and Laird random effects model was used to analyze the treatment groups. The potential presence of publication bias was examined visually by inspecting funnel plots and statistically by using the Egger’s regression model [15].

## Results

### Literature Selection and Study Characteristics

Of the 845 articles found in our initial search, 803 were excluded by screening the titles and abstracts (Figure 1). The remaining 42 articles were potentially eligible trials that examined the effects of the calcimimetic agents. Of the 42 articles, 27 were further excluded due to the reasons indicated in Figure 1. Ultimately, 15 trials were included, all of which were performed between 2000 and 2011, enrolling a total of 3387 dialysis patients [16], [17], [18], [19], [20], [21], [22], [23], [24], [25], [26], [27], [28], [29], [30].None of the conference abstracts met the inclusion criteria and therefore were not included for analysis. Multiple publications were excluded from the count of included studies because they were secondary publications of previous reports; however, any relevant and unique results were extracted and included [26], [29], [30], [31], [32], [33], [34]. Table 1 shows the characteristics of the populations and interventions of all studies included in this meta-analysis. They compared a calcimimetic agent plus standard therapy with the placebo plus standard therapy or cinacalcet plus low-dose vitamin D sterols with flexible doses of vitamin D sterols. The treatment duration ranged from 1 to 52 weeks.

**Table 1:** Characteristics of Trials of Calcimimetic Agents for SHPT in Dialysis Patients.

Reference	No. of Patients (treatment/control)	Dialysis vintage (treatment/control)	Interventions		Duration	Jadad Score
			Treatment Group (target iPTH)	Control		
Goodman et al, 2000	21(16/5)	6.76±1.0 y/8.36±1.7 y	R-568, 100 mg/d	Placebo	15d	3
Goodman et al, 2002	52 (40/12)	≥3 mo	AMG073, 5–100 mg/d	Placebo	3d single dose, 8d multiple doses, follow up 15dafter the begin	3
Quarles et al, 2003	71 (36/35)	71.3±54.3 mo/71.1±66.2 mo	AMG073, 25–100 mg/d (iPTH decrease ≥30%)	Placebo	18w (Titration, 12w; maintenance, 6w)	3
Lindberg et al, 2003	78 (39/39)	65.1±55.9 mo	AMG073, 10–50 mg/d (iPTH decrease≥30%)	Placebo	18w (Titration, 12w; maintenance, 6w)	4
Block et al, 2004	741(371/370)	72±63 mo/72±68 mo	Cin,30–180 mg/d (iPTH<250 pg/mL)	Placebo	26 w(Titration,12w; maintenance,14w)	3
Harris et al, 2004	23 (17/6)	HD	Cin, 25 mg-300mg/d	Placebo	1w of each dosing period	3
Lindberg et al, 2005	395(294/101)	56.4±53.1 mo/63.6±65.0 mo	Cin, 30–180 mg/d (iPTH <250 pg/mL)	Placebo	26w (Titration, 16w; maintenance, 10w)	4
Martin et al, 2005	410(205/205)	67±56 mo/62±55 mo	Cin, 30–180 mg/d (iPTH <250 pg/mL)	Placebo	26w	4
Sterrett et al, 2007	210(99/111)	≥3 mo	Cin, 30–180 mg/d	Placebo	52w	4
Akiba et al, 2008	109(79/30)	approximately 150 mo	Cin, 12.5 mg/d, 25 mg/d,50 mg/d	Placebo	Treatment, 3w;Follow-up, 2w	5
Fishbane et al, 2008 (ACHIEVE)	173(87/86)	46.3±36.4 mo/46.8±44.1 mo	Cin (30–180 mg/d) pluslow-dose active VitD (iPTH150–300 pg/mL)	Vit D	33w (Screening, 6w;Titration, 16w; Efficacy-assessment, 11w)	2
Fukagawa et al 2008	144(72/71)	170.4±93.7 mo/173.3±76.0 mo	Cin, 25–100 mg/d (iPTH ≤250 pg/mL)	Placebo	Screening period, 4w; Treatment period, 14w	4
Malluche et al, 2008	48 (32/16)	≥1 mo	Cin, 30–180 mg/d (iPTH ≤200 pg/mL)	Placebo with Vit Dand/or P binders	52w (Titration, 24w; maintenance, 28w)	4
Messa et al, 2008 OPTIMA	552(368/184)	64.1±72.1mo/69.4±73.6mo	Cin, 30–180 mg/d (iPTH >300 pg/ml)	Conventional care	Dose-optimization, 16w; Efficacy- assessment, 7w	2
Raggi et al, 2011 ADVANCE	360(180/180)	37.5 mo (9.3, 105.0) Median/36.7 mo (10.0, 107.5) Median	Cin (30–180 mg/d) plus low-dose active Vit D (iPTH ≤300 pg/mL)	Vit D	52w (Titration, 20w; maintenance, 32w)	2

Abbreviations: SHPT, secondary hyperparathyroidism; iPTH, intact PTH; HD, hemodialysis; Cin, Cinacalcet; Vit, Vitamin; d, day; w, week; mo, month;

The quality of the 15 included trials were assessed using the three-question instrument proposed by Jadad et al. [11] (Table 1). All 15 trials included statements regarding randomization, including seven trials that described the detailed methods used for randomization [21], [22], [23], [24], [25], [27], [28]. Thus, all trials were scored as 1 or 2 based on the randomization criteria. 12 trials that reported adequate withdrawals and drop-outs were scored as 1, while the other 3 trials were scored as 0 [22], [25], [27]. Twelve trials that reported an appropriate binding method were scored as 1–2, while the other three trials were open-label and were scored as 0 [26], [29], [30].

### Effects on Biochemical Outcomes

**Figure 2:**
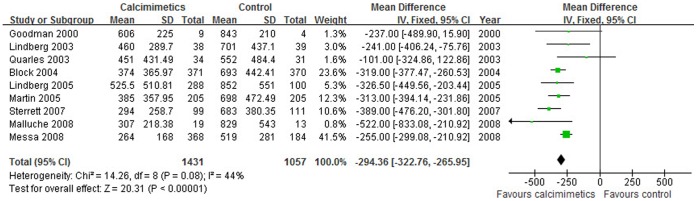
Forest plot of iPTH of patients treated with calcimimetics and control therapy. Studies are identified by name of the first author and year of publication. Mean differences (MDs) are pooled using the fixed-effect model and shown on a scale of −500 to 500.

**Figure 3:**
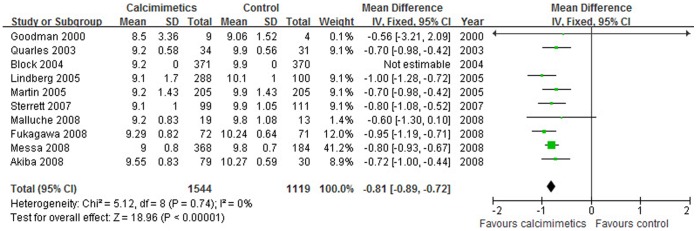
Forest plot of serum calcium of patients treated with calcimimetics and control therapy. Studies are identified by name of the first author and year of publication. Mean differences (MDs) are pooled using the fixed-effect model and shown on a scale of −2 to 2.

**Figure 4:**
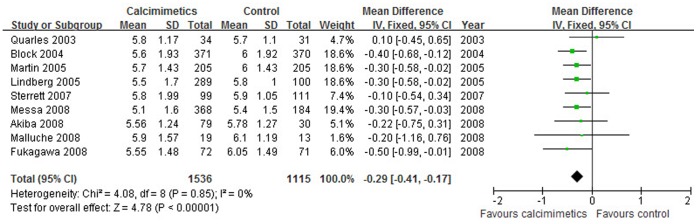
Forest plot of serum phosphate of patients treated with calcimimetics and control therapy. Studies are identified by name of the first author and year of publication. Mean differences (MDs) are pooled using the fixed-effect model and shown on a scale of −2 to 2.

The iPTH value was significantly lower in calcimimetics group than that in control therapy group (9 trials, 2488 patients; WMD, −294.36 pg/mL; 95% CI, −322.76 to −265.95, *P*<0.001; without significant heterogeneity, *P* = 0.080, I^2^ = 44%, Figure 2) and a significantly greater proportion of patients in calcimimetics group showed a ≥30% decrease in mean iPTH *vs.* the baseline as compared with the control group (OR = 10.75, 95% CI: 6.65–17.37, *P*<0.001). Similarly, serum calcium values (10 trials, 2663 patients; WMD, −0.81 mg/dL; 95% CI, −0.89 to −0.72, *P*<0.001; without heterogeneity, *P* = 0.740, I^2^ = 0%, Figure 3), serum phosphorus (9 trials, 2651 patients; WMD, −0.29 mg/dL; 95% CI, −0.41 to −0.17, *P*<0.001; with no significant heterogeneity, *P* = 0.850, I^2^ = 0%, Figure 4) and calcium phosphorus product (8 trials, 2240 patients; WMD, −7.68 mg^2^/dL^2^; 95% CI, −8.93 to −6.43, *P*<0.001; with no significant heterogeneity of these trial results, *P* = 0.470, I^2^ = 0%) were significantly lower in calcimimetics group than that in control therapy group.

There was no significant difference in bone alkaline phosphatase between the two groups (3 trials, 284 patients; WMD, 1.79 U/L; 95% CI, −7.14 to 10.71, *P* = 0.690; without heterogeneity, *P* = 0.170, I^2^ = 44%). The osteocalcin (2 trials, 252 patients; WMD, −46.85 ng/ml; 95% CI, −80.51 to −13.18; *P* = 0.006; with significant heterogeneity, *P* = 0.130, I^2^ = 55%) and tartrate-resistant acid phosphatase levels (WMD, −1.33 ng/ml; 95% CI, −2.01 to –0.65; *P* = 0.001; with no significant heterogeneity, *P* = 0.390, I^2^ = 0%) were significantly lower in calcimimetics group than those in control therapy group.

### Effects on Patient-level Outcomes

Six trials reported all-cause mortality (Table 2). Eight trials reported all adverse events including hypocalcemia, nausea, vomiting, diarrhea, dyspnea, upper respiratory tract infection, and headache. There was no significant difference in all-cause mortality and all adverse events between calcimimetics group and control therapy group (OR = 0.86, 95% CI: 0.46–1.60, *P* = 0.630; OR = 1.30, 95% CI: 0.78–2.18, *P* = 0.320, respectively). Compared with control therapy, a statistically significant increase for hypocalcemia was observed in calcimimetics group (OR = 2.46, 95% CI: 1.58–3.82, *P*<0.001). Calcimimetic therapy also led to more nausea (OR = 2.45, 95% CI: 1.29–4.66, *P* = 0.006), vomiting (OR = 2.78, 95% CI: 2.14–3.62, *P*<0.001) and diarrhea (OR = 1.51, 95% CI: 1.04–2.20, *P* = 0.030). Treatment with calcimimetic resulted in more upper respiratory tract infection, but not dyspnea or headache (OR = 1.97, 95% CI: 0.87–4.45, *P* = 0.100; OR = 1.62, 95% CI: 0.97–2.72, *P* = 0.070, respectively). The pooled ORs for all adverse events and nausea were performed using the random-effort model because of heterogeneities. There was no significant heterogeneity for other patient-level outcomes.

**Table 2:** Effect of calcimimetics and control therapy on patient-level outcomes (All-cause mortality, all adverse events, hypocalcemia, nausea, vomiting, diarrhea, dyspnea, upper respiratory tract infection and headache).

	Fixed-effects Model		Random-effects Model		Heterogeneity	
	OR (95%CI)	*P* value	OR(95%CI)	*P* value	*P* value	*I* ^2^ (%)
All adverse events	1.43 (1.14, 1.80)	0.002	1.30 (0.78, 2.18)	0.320	<0.001	74%
All-cause mortality	0.86 (0.46, 1.60)	0.630	0.86 (0.46, 1.60)	0.630	0.980	0%
Hypocalcemia	2.46 (1.58, 3.82)	<0.001	2.45 (1.11, 5.41)	0.030	0.190	32%
Nausea	2.45 (1.29, 4.66)	0.006	2.53 (2.01, 3.18)	<0.001	<0.001	79%
Vomiting	2.78 (2.14, 3.62)	<0.001	2.73 (2.07, 3.60)	<0.001	0.400	3%
Diarrhea	1.51 (1.04, 2.20)	0.030	1.49 (1.01, 2.22)	0.050	0.370	4%
Dyspnea	1.97 (0.87, 4.45)	0.100	1.93 (0.85, 4.40)	0.120	0.630	0%
Upper respiratory tract infection	1.79 (1.20, 2.66)	0.004	1.79 (1.20, 2.67)	0.004	0.480	0%
Headache	1.62 (0.97, 2.72)	0.070	1.60 (0.95, 2.69)	0.080	0.720	0%

Abbreviations:OR, Odds ratio; 95%CI, 95% confidence interval;

### Sensitivity Analysis

Further analysis of the results using the fixed effect and random effect models showed the identical results, except all adverse events which should be interpreted with caution (Table 2). Twelve of the 15 included studies compared calcimimetic agents with placebos; two trials compared cinacalcet plus low-dose active vitamin D with flexible dosing of active vitamin D [29], [30]; and the remaining trial compared cinacalcet with conventional therapy [26]. The latter three trials were open-label and low quality (Jadad score lower than 3) [26], [29], [30]. The results were similar when the three trials were excluded.

### Publication Bias

**Figure 5:**
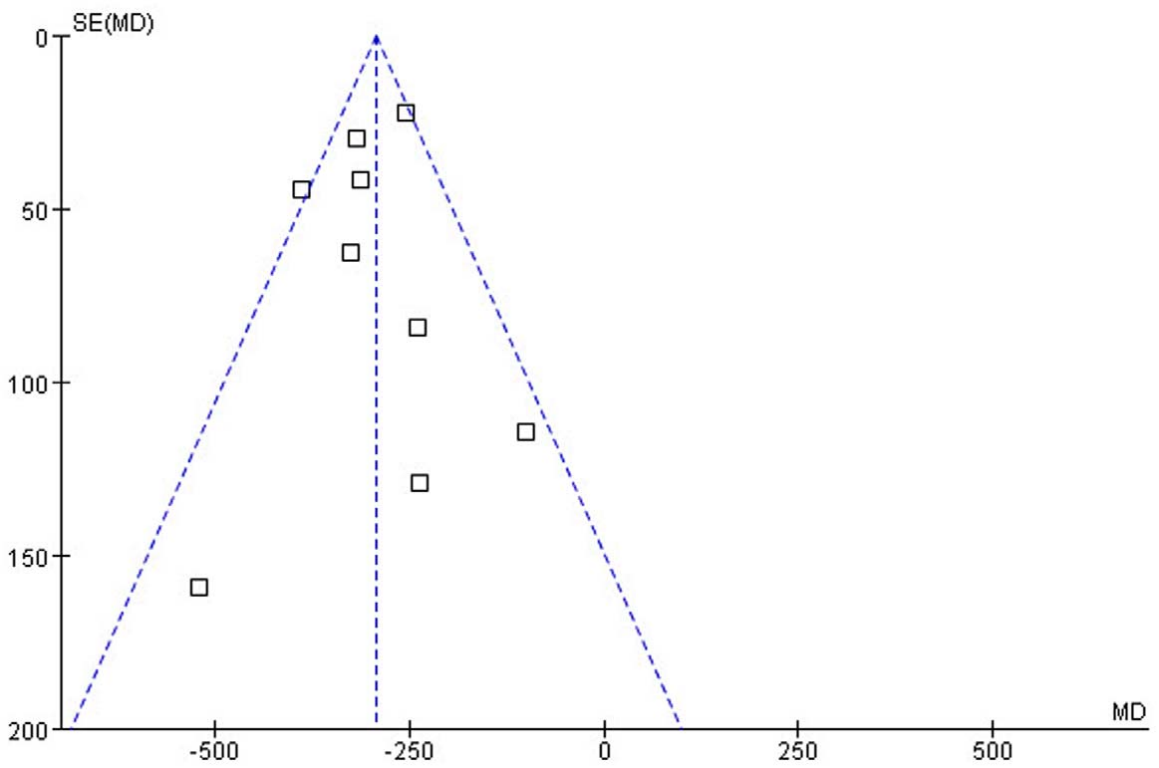
Funnel graph for the assessment of potential publication bias in iPTH. The funnel graph plots the MD against the SE of the MD. The dashed line indicates 95% confidence limits of the MD. The funnel plots show approximate symmetry. The result of the Egger’s test for publication bias was not significant (*P* = 0.724).

Publication bias was detected by using the Egger’s regression model, and the result showed that the publication bias was insignificant (*P* = 0.724). The funnel plots for publication bias (Figure 5) also showed symmetry. These results indicate that there was no publication bias.

## Discussion

A comprehensive search was performed for randomized clinical trials that evaluated the efficacy and safety profile of calcimimetic agents, and finally 15 trials involving 3387 dialysis patient with SHPT met our inclusion criteria. Our meta-analysis showed that there was no significant difference in all-cause mortality and all adverse events between calcimimetics group and control therapy group. However, there was a statistically significant increase in the episodes of hypocalcemia, nausea, vomiting, diarrhea and upper respiratory tract infection in calcimimetics group as compared with control therapy group.

Our meta-analysis also showed that calcimimetic agents effectively ameliorated iPTH levels in patients with SHPT who were undergoing dialysis and reduced serum calcium and phosphorus disturbances associated with adverse clinical outcomes. The percentage of patients in whom there was a 30% decrease in serum iPTH by the end of the dosing was higher in cinacalcet group than that in the control group. Administration of vitamin D sterols and phosphate binders in four trials [18], [21], [22], [24] was kept relatively constant to isolate the effect of cinacalcet. After excluding the four trials, we got the similar result with respect to iPTH (data not shown).

Tartrate-resistant acid phosphatase is an enzyme that is expressed in high amounts by bone resorbing osteoclasts [35], while osteocalcin is produced by osteoblasts and often used as a marker for bone formation [36]. Osteocalcin and tartrate-resistant acid phosphatase levels tended to be low in patients receiving calcimimetics, suggesting that cinacalcet may improve bone metabolism in dialysis patients. Bone alkaline phosphatase is produced by osteoblasts and osteoblast precursors, and participates in bone mineralization. High serum levels of alkaline phosphatase result from osteoclastic hyperactivity. Bone alkaline phosphatase levels were correlated with histomorphometric parameters of bone formation and bone resorption [37]. As there was no statistically significant difference in bone alkaline phosphatase levels between cinacalcet group and the control group, whether bone alkaline phosphatase is useful for estimating bone changes during cinacalcet therapy remains to be elucidated.

Previous studies showed that calcimimetic agents could suppress parathyroid hyperplasia and ameliorate osteitis fibrosa in chronic renal insufficiency models [38], [39]. In the included trials, only one trial [28] assessed the effect of cinacalcet on bone histology and showed that cinacalcet treatment reduced bone turnover and tissue fibrosis in SHPT patients receiving dialysis. Adynamic bone was also observed in three patients who received cinacalcet, and overexpression of iPTH (<100 pg/ml) was detected in two of them. It should be noted that PTH oversuppression may not be beneficial and could be associated with increased risk for low turnover bone disease. KDIGO guideline [5] suggest that if iPTH levels fall below two times the upper limit of normal for the assay, calcitriol, vitamin D analogs, and/or calcimimetics should be reduced or discontinued. Hence, iPTH levels should be closely monitored and the cinacalcet dosage should be adjusted during the treatment.

Coronary artery calcification is a common and severe problem that is associated with ischemic cardiovascular disease and mortality in adult ESRD patients [40]. Compared with control treatment, there was no evidence that cinacalcet reduced all-cause mortality and cardiovascular mortality. The ADVANCE study [29] evaluated the effects of cinacalcet plus low-dose vitamin D on vascular calcification in hemodialysis patients and demonstrated that increases in calcification scores were less in the aorta, aortic valve and mitral valve in patients treated with cinacalcet plus low-dose vitamin D sterols, suggesting that cinacalcet treatment and low-dose vitamin D sterols may attenuate the progression of established cardiovascular calcification in patients receiving hemodialysis. More clinical evidence is needed to see whether cinacalcet is associated with a survival benefit in dialysis patients.

The most commonly reported adverse events are gastrointestinal adverse events and hypocalcemia. The mechanisms of gastrointestinal adverse events remain unclear, and further basic and clinical studies are needed. Hypocalcemia is considered to result from the loss of the effects of PTH on calcium reabsorption from the distal nephron or reduced bone resorption [41]. There were a small number of drop-outs caused by hypocalcemic events, although hypocalcemia could be managed by adjustment of the cinacalcet dose or increasing the dose of calcium and/or vitamin D sterols. The long-term effect of hypocalcemia remains uncertain.

The major limitation of this meta-analysis is that the number of published studies on calcimimetic agents in dialysis patients with SHPT is limited. The duration of most of these trials ranged from 1 to 52 weeks. Three of these trials were relatively small and lasted a relatively short time [17], [18], [19]. After excluding the three trials, we got the similar result (data not shown). Only two trials [21], [26] included peritoneal patients, and therefore larger samples are required to examine the effect of cinacalcet in peritoneal patients. Nevertheless, the generalizability of all meta-analyses is limited by protocol heterogeneity and differences among study populations. In this study, the results of osteocalcin, all adverse events and nausea were considered heterogeneous. A random-effects analysis was used to account for potential differential effects across studies. In sensitivity analysis, we further analyzed the results by using both fixed effect and random effect models, and the results obtained were identical, except for all adverse events.

In conclusion, the results of this meta-analysis indicate the potential of calcimimetic agents as a treatment for dialysis patients with SHPT. Future studies are needed to assess the effects of cinacalcet on parathyroid hyperplasia, vascular calcification, bone histomorphometry, or other hard clinical outcomes in larger samples with longer durations.

## Supporting Information

Table S1 **PRISMA flow diagram of this meta-analysis.**
(DOC)

Checklist S1 **PRISMA Checklist of this meta-analysis.**
(DOC)
