# Supplementary material for: Effects and Safety of Calcimimetics in End Stage Renal Disease Patients with Secondary Hyperparathyroidism: A Meta-Analysis
Source: PLoS One. 2012 Oct 25;7(10):e48070. doi: 10.1371/journal.pone.0048070 (PMC3485048; doi:10.1371/journal.pone.0048070)
Supplement: Table S1 — PRISMA flow diagram of this meta-analysis. (DOC) [file pone.0048070.s001.doc]

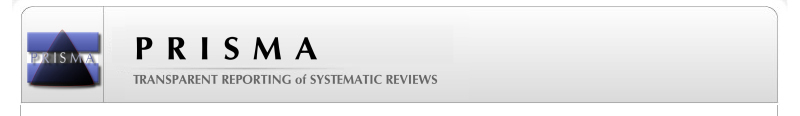
**PRISMA 2009 Flow Diagram**

**Screening**

**Included**

**Eligibility**

**Identification**

Records identified through database searching
(n = 702 )

Additional records identified through other sources
(n =143 )

Records after duplicates removed
(n = 803 )

Records screened
(n =42)

Records excluded
(n =13 )

Full-text articles assessed for eligibility
(n =29)

Full-text articles excluded, with reasons
(n =7)

Studies included in qualitative synthesis
(n =22 )

Studies included in quantitative synthesis (meta-analysis)
(n =15)
